# Regulation and roles of FOXK2 in cancer

**DOI:** 10.3389/fonc.2022.967625

**Published:** 2022-09-12

**Authors:** Yuanyuan Kang, Kexin Zhang, Lixue Sun, Ying Zhang

**Affiliations:** School and Hospital of Stomatology, China Medical University, Liaoning Provincial Key Laboratory of Oral Diseases, Shenyang, China

**Keywords:** FOXK2, post-translational modifications, forkhead box, promotive, suppresive

## Abstract

Forkhead box K2 (FOXK2) is a member of the forkhead box transcription factor family that contains an evolutionarily conserved winged-helix DNA-binding domain. Recently, an increasing number of studies have demonstrated that FOXK2 plays an important role in the transcriptional regulation of cancer. Here, we provide an overview of the mechanisms underlying the regulation of FOXK2 expression and function and discuss the roles of FOXK2 in tumor pathogenesis. Additionally, we evaluated the prognostic value of FOXK2 expression in patients with various cancers. This review presents an overview of the different roles of FOXK2 in tumorigenesis and will help inform the design of experimental studies involving FOXK2. Ultimately, the information presented here will help enhance the therapeutic potential of FOXK2 as a cancer target.

## Background

Cancer is one of the major causes of death worldwide (1). In 2018, there were 18.1 million new cancer cases and 9.6 million cancer-related deaths (2). *Cancer Tomorrow* predicts that by 2040, there will be a 63.1% increase in total new cancer cases over 2018 levels (3). Cancer is a major cause of morbidity and mortality worldwide (4), and despite significant progress in diagnostics and treatments, it remains a critical health concern. This may be due to the unknown aspects of carcinogenic mechanisms, including the role of transcription factors.

The forkhead box (FOX) family of transcription factors originates in unicellular eukaryotes and is named after the *Drosophila melanogaster* (fruit fly) gene forkhead (fkh) (5). The specificity of FOX proteins is determined by the highly conserved FKH DNA-binding domain, which comprises approximately 100 residues (6). Based on the conserved DNA-binding domain, the FOX family of proteins can be divided into 19 sub-families: FOXA to FOXS (7). FOXK transcription factors are widely expressed in various tissues and play crucial roles in the cellular functions of higher organisms. The FOXK family includes two members, FOXK1 and FOXK2, which can regulate metabolism, cell cycle progression, proliferation, survival, differentiation, and apoptosis (8). All functions are mediated by the specific activation of a coordinated transcriptional program. The role of FOXK1 in tumors has been thoroughly studied. Thus, in this review, we summarize the latest information regarding the molecular mechanisms by which FOXK2 expression and activity are regulated and the role of FOXK2 in cancer. Additionally, we focused on the prognostic value of FOXK2 expression in various cancers.

## Structure and function of FOXK2

**Figure 1:**
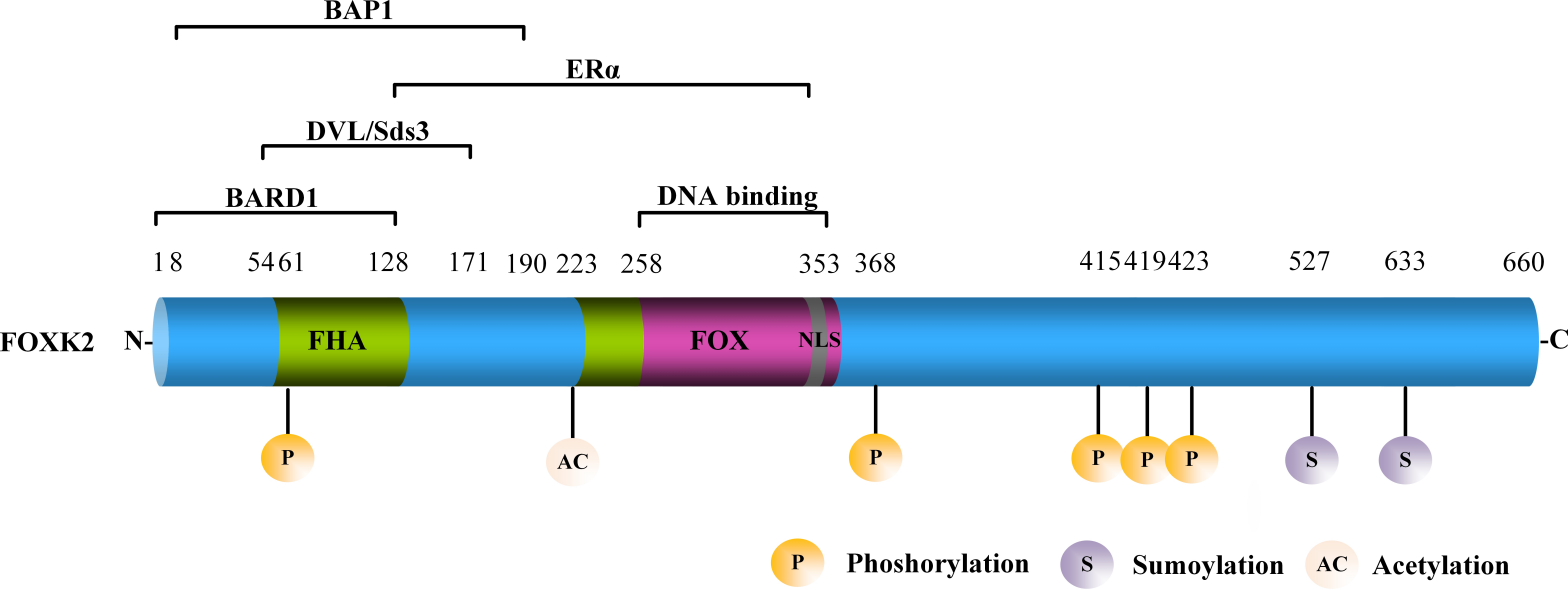
The structure of FOXK2. FOXK2 comprises a FHA, FOX domain, and NLS. Well-known proteins that interact with FOXK2 are shown above the corresponding domains. The post-translational modification (PTM) sites of FOXK2 are shown.

FOXK2, also known as ILF or ILF1, is located on human chromosome17q25.3, which encodes a functional FOXK2 protein containing 660 amino acids (9). FOXK2 is characterized by a forkhead-associated (FHA) domain, a FOX domain that mediates its interaction with DNA, and a nuclear localization signal (NLS) (**Figure 1**). The FHA domain of FOXK2 mediates interaction with other proteins. For example, the amino acid region 1–128 mediates the interaction between FOXK2 and the breast cancer gene 1 (BRCA1) ring domain 1 (BARD1) (10). The amino acid region 54–171 mediates the interaction between FOXK2 and disheveled (DVL) or Sds3 (11, 12). The amino acid region 128–353 mediates the interaction between FOXK2 and estrogen receptor α (ERα), leading to lower ERα protein stability and inhibition of its transcriptional activity *via* a mechanism that involves BRCA1/BARD1 (10). The amino acid region 8–190 primarily mediates the interaction between FOXK2 and BAP1 (13) (**Figure 1**). FOXK2 mRNA undergoes alternative splicing with three isoforms identified to date (14, 15), whose functional differences have not been explored.

## FOXK2 expression and cell metabolism

Although FOXK2 was discovered in the early 1990s, its biological role has remained elusive for some time. Only within the past decade have the many broad and unique functions of FOXK2 been discovered, including its participation in various molecular signaling pathways.

### FOXK2 and autophagy

**Figure 2:**
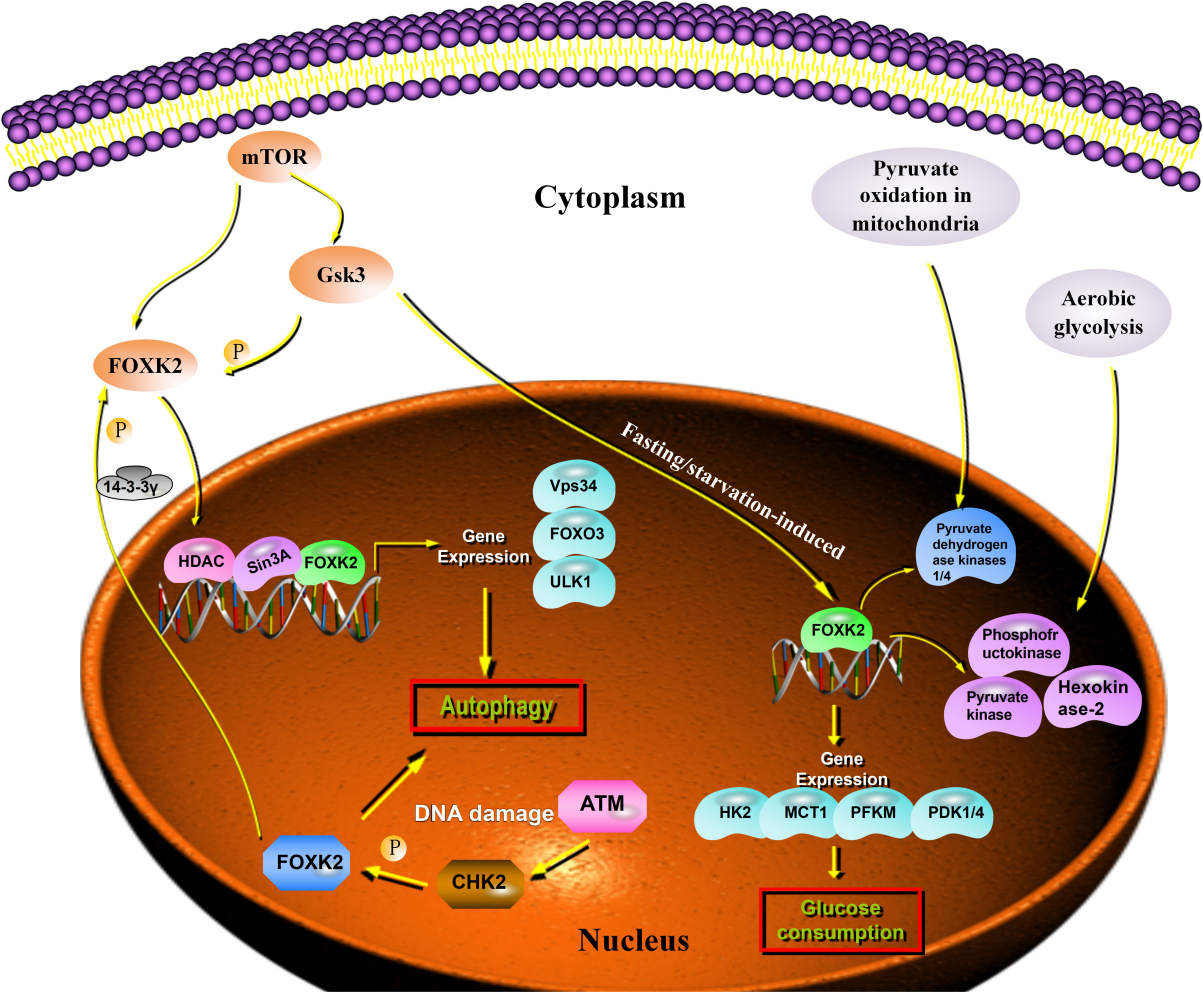
FOXK2 expression and cell metabolism. Autophagy genes are inhibited through the direct binding of FOXK2 and recruitment of Sin3A–HDAC complexes, resulting in the reduction of activating H4ac marks. FOXK2 is regulated by the downstream of mTOR. Upon DNA damage, ATM activates CHK2, which directly phosphorylates FOXK2 and creates a 14-3-3γ binding site. This site, in turn, traps FOXK2 in the cytoplasm. mTOR induces nuclear translocation of FOXK2 through GSK regulation and FOXK2 induces glucose consumption. In addition, FOXK2 regulates aerobic glycolysis and inhibits aerobic oxidation by upregulating glycolysis target genes.

Autophagy is a self-degradation process that plays an important role in balancing energy sources and eliminating harmful metabolites such as misfolded proteins and reactive oxygen species (16, 17). Recent studies have shown that FOXK2 functions as a transcriptional inhibitor of autophagy. Interestingly, FOXK2 was used to balance the activity of another forkhead transcription factor, FOXO3, which induces a set of overlapping autophagy and atrophy targets in muscle. FOXK2 also limits the expression of autophagy genes by recruiting Sin3A-HDAC complexes. Notably, under nutrient-rich conditions, the mammalian target of rapamycin (mTOR) phosphorylates and activates FOXK2 to limit basal levels of autophagy (18). Chen demonstrated that the FOXK2 protein plays an important role in transcriptional regulation, linking DNA damage and autophagy. DNA damage induces CHK2-mediated FOXK2 phosphorylation at Ser61 and traps FOXK2 in the cytoplasm by binding to 14-3-3γ (**Figure 2**). Trapping FOXK2 in the cytoplasm relieves the inhibition of autophagy-related genes (ATGs) and promotes autophagy (19) (**Figure 2**). The autophagy-related proteins, ULK1, Vps34, and FOXO3, were markedly upregulated by FOXK2 knockdown and downregulated by FOXK2 overexpression. Although FOXK2 can inhibit autophagy, tumor cells can relieve the inhibitory effect of FOXK2 on autophagy through various mechanisms to protect their malignant behavior under conditions of nutritional deficiency, radiotherapy, chemotherapy, and stress.

### FOXK2 and aerobic glycolysis

A series of tumor-metabolism related genes are regulated by FOXK2 at the transcriptional level. Some studies have demonstrated that fasting/starvation-induced FOXK2 induces aerobic glycolysis by upregulating the required enzymatic machinery (including hexokinase-2, phosphofructokinase, pyruvate kinase, and lactate dehydrogenase), while simultaneously suppressing further oxidation of pyruvate in the mitochondria by increasing the activity of pyruvate dehydrogenase kinases 1 and 4 (20). FOXK2 was upregulated in the muscle and adipose tissue of wild-type C57BL/6J mice that were starved for 16 h, and in skeletal muscle after exercise. In response to FOXK2 overexpression, glucose uptake increased in myoblasts and myotubes, an effect that was reversed by FOXK2 knockdown. Additionally, FOXK2 overexpression induced glycolysis in adipocytes, suggesting that it is a key transcriptional regulator of glycolytic enzymes. Interestingly, transcriptome analysis of adipocytes either overexpressing or lacking FOXK2 revealed that differentially expressed glycolytic pathway genes were co-regulated by FOXK2, indicating additional redundancies in their functions (20). The *in vivo* relevance of these findings has been confirmed, as mice lacking FOXK2 expression exhibited reduced glucose uptake, supporting a role for FOXK2 in regulating aerobic glycolysis (**Figure 2**).

## Molecular mechanisms of FOXK2 regulation

Emerging evidence suggests that the there are several different mechanisms involved in the regulation of FOXK2 activity in cancer cell, such as hypermethylation, noncoding RNAs (ncRNAs), and post-translational modifications (PTMs). Here, we introduce several important regulatory modes of FOXK2 in cancer.

### Transcriptional regulation of FOXK2 by DNA methylation

Hypermethylation of promoter regions inhibits gene expression, and genome-wide hypomethylation leads to genome instability and the activation of oncogenes (21). In mouse embryonic stem cells, FOXK2 binds methylated DNA (22). Park et al. found that urinary nicotine equivalents are significantly associated with FOXK2-methylated CpG sites (23). Although the role of DNA methylation in transcriptional regulation is still unclear, a report by Baymaz et al. showed that FOXK2/polycomb repressive deubiquitinase (PR-DUB) and MBD6 share a subset of genomic target genes, establishing a link between DNA methylation and the recruitment of transcriptional complexes (24). In this study, MBD5 and MBD6 interacted with PR-DUB, and MBD6 was recruited to the site of DNA damage after microirradiation in a PR-DUB-independent manner. In conclusion, these studies demonstrated that FOXK2 was involved in DNA methylation.

### Transcriptional regulation of FOXK2 by ncRNAs

Several studies have revealed that ncRNAs function as tumor regulators by targeting transcription factors, including FOXK2 (**Table 1**). MicroRNAs (miRNAs) are a group of small, single-stranded ncRNAs (18–25 nucleotides in length) that regulate gene expression by binding to the 3’UTR of the corresponding target mRNA (31). By targeting FOXK2, miR-602 promotes esophageal squamous cell carcinoma proliferation and metastasis and regulates cell cycle progression. More importantly, in nude mice, systemic delivery of the planned miR-602 antagomir reduces tumor growth and increases FOXK2 protein expression (25). In hepatocellular carcinoma (HCC), FOXK2 is a direct target of miR-1271. Re-introduction of miR-1271 decreased FOXK2 mRNA and protein levels. FOXK2 expression was also reported to be inversely correlated with miR-1271-5p expression in HCC samples (26). Additionally, the expression of FOXK2 may be regulated by miR-1271 in non-small-cell lung cancers (28).

**Table 1:** ncRNAs targeting FOXK2 in cancer.

Cancer types	ncRNA	Function	Ref.
Esophageal squamous cell carcinoma	miR-602	Promoted the proliferation, metastasis and regulated cell cycles.	(25)
Hepatocellular carcinoma	miR-1271-5p	FOXK2 expression was reversely connected with miR-1271-5p in clinical samples.	(26)
	LncRNA SNHG7 miR-122-5p	Promoted the cell growth and metastasis. Inhibited the cell growth and metastasis.	(27)
Non-small-cell lung cancer	miR-1271	FOXK2 was negatively targeted by miR-1271 in cells.	(28)
Cervical cancer	Circ-ITCH miR-93-5p	Inhibited the proliferation, migration, and invasion of cells. Promoted the proliferation, migration, and invasion of cells.	(29) (29)
Clear cell renal cell carcinoma	CircUBAP2 miR-148a-3p	Inhibited the proliferation, migration, and invasion of cells. Promoted the proliferation, migration, and invasion of cells.	(30) (30)

Circular RNAs (circRNAs) are a special group of RNAs consisting of hundreds of nucleotides that regulate gene expression at the transcriptional or post-transcriptional level by binding to target miRNAs (32). In cervical cancer, circ-ITCH sponges miR-93-5p and upregulates FOXK2 by suppressing cell proliferation, migration, and invasion (29). Aberrant expression of circ-UBAP2 inhibits the proliferation, migration, and invasion of clear cell renal cell carcinoma (ccRCC) cells by sponging miR-148a-3p. The promotion of cell proliferation and invasion by the restoration of FOXK2 expression in circ-UBAP2 knockdown cells confirmed the circ-UBAP2-miR-148a-3p-FOXK2 axis in ccRCCs (30).

Long noncoding RNAs (lncRNAs), which are usually divided into five categories, are primarily transcribed by RNA polymerase II and lack an obvious open reading frame (33). Like miRNAs and circRNAs, lncRNAs can regulate the expression of FOXK2. For example, the lncRNA SNHG7 enhances the development of HCC by sponging miR-122-5p to regulate FOXK2 (27). This finding suggests that lncRNAs and FOXK2 are involved in the occurrence and development of cancer.

### Post-translational regulation of FOXK2

PTMs regulate protein structure and function by covalently adding functional groups, peptides, or other complex molecules to extend and diversify protein properties (34, 35). To date, more than 300 types of PTMs have been identified (36). Accumulating evidence indicates that the functions of FOXK2 are altered in cancers by PTMs, which affect the expression of FOXK2 target genes (**Table 2**).

**Table 2:** Regulation of FOXK2 through PTMs and their functions.

Type of PTMs	Sites	Biological functions	Ref.
Phosphorylation	Ser61 Ser368 Ser423 Ser415 Ser419	DNA damage induces CHK2-mediated FOXK2 phosphorylation and traps FOXK2 in the cytoplasm through binding with 14-3-3γ. Promotes apoptosis and FOXK2 is linked to the cell cycle regulatory machinery. Translocation of FOXK2 transcription factors from cytoplasm to nucleus, and modulated in a ligand-dependent and receptor-dependent manner.	(19) (37) (38)
Deubiquitination	K119	FOXK2 binds to the SIN3A and PR-DUB complexes, which contains the deubiquitinase BAP1. FOXK2 recruits BAP1 to DNA, promotes local histone deubiquitination and causes changes in target gene activity.	(39)
Sumoylation	Lys527 Lys633	SUMOylation might act to enhance FOXK2 transcriptional activity and mediate the cytotoxic response to paclitaxel through the tumour suppressor FOXO3 in breast cancer.	(40)
deacetylation	K223	SIRT1 mediated the deacetylation of FOXK2.	(41)

**Figure 3:**
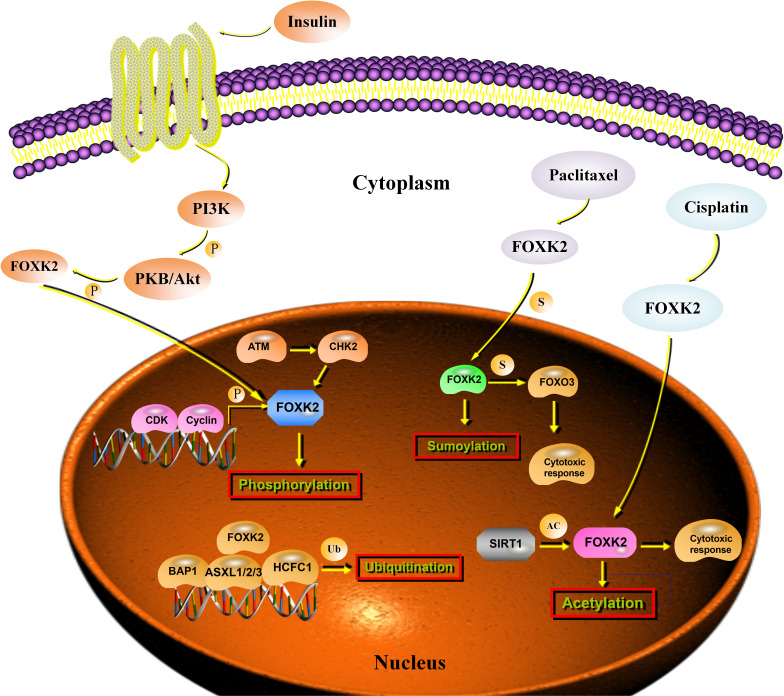
Post-transcriptional regulation of FOXK2. Stimulation with insulin results in the phosphorylation of FOXK2 through PI3K-Akt that leads to the translocation of FOXK2. CDK/cyclin complexes phosphorylate FOXK2 and promote its degradation. ATM activates CHK2, which phosphorylates FOXK2 at Ser61. BAP1 binding to chromatin depends on FOXK2. A PR–DUB complex is produced, which regulates histone H2AK119ub1 levels and transcription. Sumoylation of FOXK2 increases its transcriptional activity and mediates the cytotoxic response to paclitaxel through FOXO3a. SIRT1 inhibition enhanced FOXK2-induced chemosensitivity to cisplatin *via* acetylation.

Phosphorylation is one of the most widely studied PTMs and orchestrates various cellular functions (42). CHK2 specifically phosphorylates FOXK2 at Ser61 in response to DNA damage and induces FOXK2 binding to the 14-3-3γ protein, which, in turn, traps FOXK2 in the cytoplasm (19). Simultaneously, FOXK2 phosphorylation leads to chemo-resistance in cancer cells by modulating autophagy (19). During the cell cycle, the phosphorylation level of FOXK2 increases periodically, and hyperphosphorylation occurs in mitotic cells. Cyclin/CDK complexes are important for generating the hyper-phosphorylated form of FOXK2 during G2-M. Ser368 and Ser423 were identified as the main binding sites for FOXK2 phosphorylation by CDK-CLNA complexes (37). Insulin signaling modifies phosphorylation at Ser415/Ser419 sites, resulting in the reciprocal translocation of FOXK2, which depends on the Akt, mTOR, and GSK3 pathways (38) (**Figure 3**).

Ubiquitination and deubiquitination are dysregulated in cancer (43). FOXK2 binds to the SIN3A and PR-DUB complexes, which contain an important tumor suppressor protein, the deubiquitinase BAP1. FOXK2 recruits BAP1 to DNA, promotes local histone deubiquitination, and alters target gene activity (44). The binding of PR-DUB to target genes depends on FOXK2, and loss of the complex leads to increased H2AK119ub1 levels at target genes and their subsequent downregulation (39) (**Figure 3**).

Similar to ubiquitylation, sumoylation is a highly conserved enzymatic cascade in which a SUMO protein is enzymatically conjugated to the ϵ-amino group of certain lysine residues (45). Unlike ubiquitin modification, SUMO modification is primarily responsible for modifying substrates rather than directly degrading them (46). Recently, sumoylation (Lys 527 and Lys633) was associated with the regulation of FOXK2 activity, and its role in mediating the cytotoxic response to paclitaxel through the tumor suppressor FOXO3 in breast cancer cells was recently reported (40) (**Figure 3**). Unfortunately, there have been few studies on FOXK2 sumoylation. Hence, the precise sumoylation sites on FOXK2 and the biological mechanism of FOXK2 sumoylation require further study.

Acetylation is another reversible form of PTM that plays a key role in regulating gene expression and controlling cellular processes (47). It was not until recently that Wang et al. provided the first evidence that SIRT1 mediates the deacetylation of FOXK2. In this study, SIRT1 inhibition enhanced FOXK2-induced chemosensitivity to cisplatin *via* acetylation at K223 of FOXK2, and FOXK2 K223 deacetylation reduced the chemosensitivity of cancer cells to cisplatin (41) (**Figure 3**). Further investigations are needed to shed light on the roles of FOXK2 acetylation at other sites and determine how they crosstalk with other PTMs.

## FOXK2 in cancer progression

Studies have shown that the abnormal regulation of FOXK2 can suppress or promote the occurrence and development of certain cancers. Additionally, FOXK2 participates in the regulation of a series of biological behaviors, such as tumor metabolism, autophagy, proliferation, invasion, and metastasis, *via* multiple signal pathways. Here, we discuss the suppressive and promotive roles of FOXK2 in the context of cancer.

### Tumor-suppressive role of FOXK2

During the progression of breast cancer, the interaction of FOXK2 with ERα leads to decreased stability and ubiquitin-mediated degradation of ERα, which depends on BRCA1/BARD1 (10). Corroborating these data, a separate study reported that FOXK2 mediates the cytotoxic effects of chemotherapeutic agents (paclitaxel and epirubicin) on breast cancer cells (48). Some studies have shown that the expression of FOXK2 is gradually lost during breast cancer development. To further explore FOXK2 functions in breast cancer tumorigenesis, Shan et al. reported that FOXK2 interacts with the transcription co-repressor complexes NCoR/SMRT, SIN3A, NuRD, and REST/CoREST to repress a cohort of genes, including HIF1β and EZH2, and to regulate several signaling pathways, including the hypoxic response (49). The authors found that FOXK2 inhibited the proliferation and invasion of breast cancer cells and suppressed the growth and metastasis of breast cancer. Notably, FOXK2 is transactivated by ERα and transrepressed through reciprocal successive feedback by HIF1β/EZH2. Additionally, combining transcription inhibitory complexes, such as SIN3A, NCoR/SMRT, NuRD, and FOXK2, inhibits the transcription of a series of oncogenes, including HIF1, EZH2, and VEGFR. This affects a series of signaling pathways, including the cell cycle, DNA damage response, hypoxia response, p53 signaling, and the epithelial–mesenchymal transition (EMT), ultimately inhibiting the occurrence and development of breast cancer.

Similar to its role in breast cancer, FOXK2 has been implicated in suppressing tumorigenesis in non-small-cell lung cancer (NSCLC). FOXK2 regulates NSCLC cell growth by suppressing the expression of cyclin D1 and CDK (28). Additionally, the capacity for invasion and EMT progression of NSCLC cells were impaired following FOXK2 knockdown. Wang et al. analyzed the expression of FOXK2 in 151 patients with glioma and confirmed that the expression of FOXK2 was negatively correlated with tumor grade and prognosis (50). At the cellular level, FOXK2 inhibits the proliferation, invasion, migration, and upregulation of E-cadherin and α-catenin, and downregulates the expression of N-cadherin and vimentin, suggesting that FOXK2 modulates the expression of epithelial and mesenchymal markers closely linked to the EMT process (50). Consistent with these findings, Liu et al. reported that FOXK2 downregulation in gastric cancer cell lines inhibits cell proliferation and colony formation, in addition to the suppression of cellular migration and invasion (51). Zhang indicated that FOXK2 inhibits the malignant phenotype of ccRCC and acts as a tumor suppressor, possibly through the inhibition of EGFR (52). Interestingly, methylated FOXK2 has been observed in circulating leukocytes of smokers in an epigenome-wide association study of nicotine equivalents (23), suggesting that epigenetic modifications serve as additional mechanisms for silencing FOXK2 gene expression, which may have implications for the pathogenesis of lung cancer.

### Tumor-promotive role of FOXK2

Despite its role as a tumor suppressor, some reports have attributed an oncogenic role to FOXK2. In HCC, overexpression of FOXK2 results in enhanced cell growth and migration. Further investigations revealed that FOXK2 facilitates the development of HCC *via* the activation of the PI3K/AKT pathway; overexpression of FOXK2 resulted in a significant increase in the expression of phosphorylated Akt, survivin, c-Myc, p27, and CyclinD1 proteins, which was reversed following FOXK2 knockout. Treatment with LY294002, an inhibitor of the PI3K/AKT pathway, markedly attenuated the cell growth induced by FOXK2 in HCC cell lines (26). Another study reported that FOXK2 downregulation inhibited cell proliferation, colony formation, migration, and invasion in HCC cells and suppressed EMT partly through the inhibition of the Akt signaling pathway (53). The gene targets involved in mediating the oncogenic effects of FOXK2 in HCC remain to be identified. In addition to HCC, FOXK2 acts as an oncogene in colorectal cancer (CRC). FOXK2 promotes CRC metastasis by activating EGFR and ZEB1 (54). FOXK2 protein levels are elevated in CRCs and correlated with DVL nuclear localization, and they positively regulate Wnt/β-catenin signaling by translocating DVL into the nucleus (11). This is consistent with a report on the oncogenic role of FOXK1 in CRC (55). Corroborating these data, Qian et al. have reported that FOXK2 promotes the proliferation of CRC cell lines and that the FOXK2 promoter is transcriptionally regulated by SOX9 oncogenic proteins (56).

In papillary thyroid cancer, stable knockdown of FOXK2 markedly inhibited cell proliferation, significantly increased the ratio of LC3-II/LC3-I, and reduced p62 expression, whereas overexpression of FOXK2 resulted in the opposite phenotype (57). FOXK2 positively regulates VEGF and VEGFR-signaling networks. Functional experiments have demonstrated that FOXK2 promotes angiogenesis by inducing VEGF-A transcription. We also found that FOXK2-induced VEGF-A could bind to VEGF-R1 to compensate for VEGF-R2 blockage, which promoted angiogenesis by activating ERK, PI3K/AKT, and P38/MAPK signaling in human umbilical vein endothelial cells (58).

## Clinical significance of FOXK2 in cancer

The identification of specific and sensitive biomarkers is important for early cancer detection and for designing individualized treatment strategies for cancer patients. Molecular biomarkers highlight biological differences between cancers and help to prognosticate the outcomes of patients. During the last few decades, some studies have reported FOXK2 expression in cancer patients. In breast cancer, 53 breast tumor specimens (27 ERa-positive and 26 ERa-negative) were analyzed using immunohistochemical assays, and a negative correlation between ERa and FOXK2 was observed. FOXK2 nuclear expression correlates with FOXO3a expression and clinical outcomes in patients with breast cancer. Further research suggests that constitutively high expression levels of nuclear FOXK2 are associated with drug resistance and poor clinical outcomes (59). Shan et al. reported that the level of FOXK2 expression was negatively correlated with the histological grades of tumors, suggesting that FOXK2 expression is progressively lost during cancer progression (49). Clinical data have demonstrated that FOXK2 expression is reduced in gastric cancer tissues, and low FOXK2 expression is indicative of poor prognosis. Data obtained from the Human Protein Atlas have revealed that gastric cancer patients with high levels of FOXK2 experienced longer Overall Survival (OS) times (51). Similar to the case in gastric cancer, FOXK2 expression gradually decreased with increasing glioma grades, and low FOXK2 was indicative of poor prognosis (50). Additionally, FOXK2 protein and mRNA levels were upregulated in HCC. Most importantly, HCC patients with high FOXK2 protein expression exhibited worse survival in a multivariate analysis, indicating that FOXK2 may serve as an independent predictor of survival. In ccRCC, FOXK2 mRNA and protein levels were downregulated, and low FOXK2 expression was associated with worse disease-free survival, highlighting the potential of FOXK2 as an independent prognostic marker (60). FOXK2 is significantly upregulated in human CRC tissues and correlates with more aggressive features, again indicating poor prognosis (54). FOXK2 expression is associated with chemoradiotherapy resistance and poor prognosis in patients with locally advanced rectal cancer (LARC), and is a promising LARC biomarker (59).

Overall, there is a complicated picture emerging where FOXK2 expression may have prognostic or diagnostic value. The value of FOXK2 as a biomarker is likely highly tumor-specific, and even then may only apply to specific sub-types of specific tumors or to particular therapeutic regimes (**Table 3**).

**Table 3:** Functional roles of FOXK2 pathway in different types of cancer.

Cancer types	Function	Oncogeneor Suppressor	Ref.
Breast cancer	FOXK2 suppress ERa-mediated proliferation of cells. FOXK2 inhibited the transcriptional activity of Era via a mechanism that involved BRCA1/BARD1.	Suppressor	(10)
	SUMOylation positively regulates FOXK2 transcriptional activity and has a role in mediating the cytotoxic response to paclitaxel through the tumour suppressor FOXO3.		(45)
Non-small cell lung cancer	FOXK2 suppresses EMT and invasion by targeting N-cadherin and Snail, suppresses the activity of PI3K/AKT/mTOR signaling pathway to inhibit cell tumorigenicity.	Suppressor	(49)
Glioma	FOXK2 inhibits the proliferation and invasion of cells and suppresses the growth and metastasis of breast cancer. FOXK2 is transactivated by ERa and trans-repressed via reciprocal successive feedback by HIF1b/EZH2.	Suppressor	(48)
Glioma	FOXK2 inhibits tumor proliferation, migration, and invasion and EMT process.	Suppressor	(20)
Gastric cancer	FOXK2 inhibits the proliferation, migration and invasion of cells and induced early apoptosis.		(50)
Clear-cell renal cell carcinoma	FOXK2 suppressed the capabilities of proliferation and motility and promoted apoptosis via suppression of EGFR.	Suppressor	(51)
	FOXK2 are independent prognostic factors for patients with ccRCC.		(52)
	FOXK2 promoted cell growth indicates unfavorable prognosis.		(27)
Hepatocellular Carcinoma	FOXK2 downregulation could inhibit cell proliferation and colony formation and suppress migration and invasion. Suppressed the EMT through inhibition of the Akt signaling pathway.	Oncogene	(54)
	FOXK2-mediated DVL nuclear translocation in Wnt signaling		(11)
Colorectal cancer	FOXK2 overexpression promoted cell migration, invasion and promoted metastasis by EGFR and ZEB1, and it was induced by EGF/ ERK/NF-κB signaling.	Oncogene	(55)
	FOXK2 promoted cell growth and could be transcriptionally activated by SOX9.		(57)
Thyroid carcinoma	FOXK2 downregulation could inhibite cell proliferation, increase the ratio of LC3-II/LC3-I, and reduce p62 expression, whereas overexpression of FOXK2 showed the opposite effects	Oncogene	(58)
	FOXK2 was upregulated in ATC tissues, and the expression of FOXK2 was associated with tumor size, it promoted angiogenesis by inducing the transcription of VEGFA.		(59)

## Summary and future perspectives

FOXK2, a forkhead box transcription factor, is an important regulator of multiple physiological and pathological processes. FOXK2 plays a role in regulating the expression of a series of downstream target genes and affects multiple cancer signaling pathways. Deregulation of FOXK2 results in the initiation, development, and progression of cancer. The FOXK2 protein is regulated by many signaling networks. In many human cancers, the complex regulatory networks controlling FOXK2 activity are often significantly altered. Thus, FOXK2 exhibits great potential as a therapeutic target for various cancers. While our understanding of FOXK2 regulation during cancer development has made significant progress in recent years, there are still many puzzles that remain to be solved. Firstly, the expression, prognostic value, and clinical application of FOXK2 in different tumors require additional clinical data support. Moreover, the mechanisms underlying the activity of FOXK2 require further elucidation. The discovery of additional broad-spectrum FOXK2 regulatory targets will be of great significance for elucidating tumorigenesis mechanisms. Importantly, there have been a few clinical trials targeting FOXK2 for developing cancer therapeutics. Future research should focus on the missing links between FOXK2 activity and cancer, as such studies are likely to contribute novel insights into the mechanisms regulating carcinogenesis, which may render FOXK2 a powerful target for anti-tumor intervention therapy.

## Author contributions

YK and KZ wrote the paper. YZ and LS revised the paper. All authors collected the data and read and approved the final manuscript.

## Funding

This work was supported by the Natural Science Foundation of China (82002886), the Natural Science Foundation of Liaoning Province (2021-BS-122), and the Shenyang Science and Technology Plan Fund Project (21-173-9-40).

## Conflict of interest

The authors declare that the research was conducted in the absence of any commercial or financial relationships that could be construed as a potential conflict of interest.

## Publisher’s note

All claims expressed in this article are solely those of the authors and do not necessarily represent those of their affiliated organizations, or those of the publisher, the editors and the reviewers. Any product that may be evaluated in this article, or claim that may be made by its manufacturer, is not guaranteed or endorsed by the publisher.
